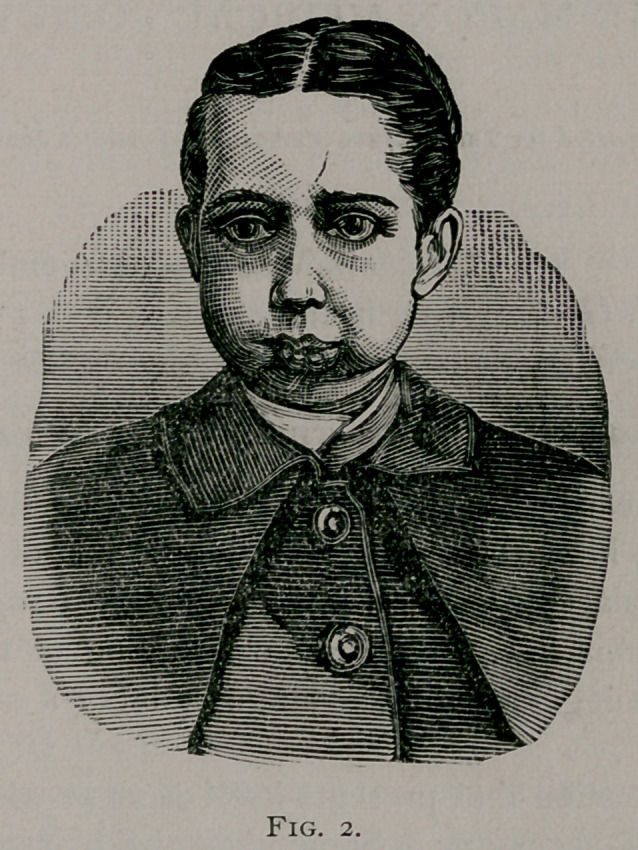# Removal of a Giant Cell Sarcoma of the Lower Jaw

**Published:** 1887-02

**Authors:** K. C. Divine

**Affiliations:** Atlanta, Georgia


### REMOVAL OF A GIANT-CELL SARCOMA OF THE LOWER JAW.

BY K. C. DIVINE, M. D., ATLANTA, GEORGIA.

On February 9th, 1886, with the assistance of Drs. Geo. H. Noble, C. L. Wilson, Hunter P. Cooper, J. McF. Gaston and Powell Walker, of Atlanta, and Dr. G. A. Hill, of Alabama, I operated upon Mrs. C. R., of Alabama. She gave the following history: Age 38 years, married and mother of six children, eldest being 16 years old and youngest 17 months; no hereditary taint. In 1873 a small growth, about the size of a filbert, appeared on the left side of the chin, which remained stationary until 1876, when it nearly disappeared under the local use of tincture iodine. It afterwards increased slowly until 1885, when it grew rapidly, with some pain, and in the course of twelve months had quadrupled in size.

The tumor presents the characteristics of sarcoma and involved the bony structure of the chin, extending back on each side to the body of the bone. It gave the following measurements: Antero-posterior diameter 3 inches, bilateral 3½ inches, vertical 4 inches, and was hard to the touch, with adhesion of the skin to the most prominent part.

After inducing complete anesthesia, the patient was operated upon as follows:

The lip being lifted by an assistant, a curved incision was made below it and through to the line of the gums, passing back on each side of the tumor; another curved incision beneath the prominence met that made previously at their extreme ends, so that an elliptical portion of the adherent skin was included between them; from the angles on each side incisions were extended back along the lower jaw. Blood flowed freely from each of these incisions on account of the abnormally developed vessels, and required to be controlled by serrefine as the operation proceeded. The skin was dissected off from the inferior portion of the tumor and at the sides until a portion of the bone was reached, which was free from disease, just in advance of the base of the coronoid process and ramus; the chain-saw was then passed and the body of the bone divided on each side. The facial artery was not cut, but several vessels required ligation. A thread was now passed through the tongue and held by an assistant, while the bony mass was depressed, and a blunt in-

FIG. 1.

strument with the fingers were used to separate it from the base of the tongue and other soft parts, leaving quite a cavern when it was detached. There was some oozing from the exterior surface which ceased upon the application of sponges wrung out of hot water, and the wound was cleansed with Gross' antiseptic mixture, consisting of hydr. bichl., gr. xx.; glycerine, ℥ jss, alcohol to make Oi., of which one fluid drachm to the quart of water was employed for washing the parts. The margins were brought together below in the median line by five stitches, and by four on each side, leaving a central opening for drainage, and the wound was covered with antiseptic cotton. The thread through the tongue was secured by adhesive plaster so as to avoid its retraction.

Upon examination of the general condition of the patient, after discontinuing the anesthetic, the influence of shock, demanded a recourse to hypodermic injections of whisky, and also of morphia with atropia, and the use of bottles of hot water to the inner surface of thighs, armpits, feet and back. In the course of three hours she had rallied completely and rested well during the night. February 10th, 8 a. m., tem. 101½, p. 120, cheerful and takes milk punch by the spoonful; 8:30 p. m., tem. 105, p. 130, complains of pain in the glenoid articulations. Gave hypodermic of sulph. morphia ¼ gr. and atropia $\frac{1}{100}$. At 9:30 p. m. patient was easy and rested well during night.

February 11th, 7 a. m., tem. 101½, p. 108. At 10 a. m. the thread was removed from the tongue. Complains of some pain, and I repeated injection of morphine and atropia; 4 p. m., tem. 100, p. 85; 10 p. m., tem. 100¼, p. 100. Pain in glenoid articulations, called for repetition of hypodermic injection.

February 12th, 8 a. m., tem. $101\frac{3}{5}$, p. 108. Has not rested well during night. The iron-dyed silk stitches, excepting one on each side, were removed from the wound and the parts found adherent with but slight suppuration. Five p. m., tem. $103\frac{4}{5}$, p. 120 and feeble with chilly sensations. The hypodermic of morphia and atropia repeated in same form.

February 13th, 7 a. m., tem. 104, p. 120. An erysipelatous blush was found on the left cheek, and lobe of ear slightly swollen. Ordered 35 drops of muriated tincture of iron with 4 grs. of quinine sulphate every 4 hours. After the lapse of 24 hours it was discovered that by a mistake of the nurse only 5 drops of the iron had been given with each dose of the quinine; and in the meantime the erysipelas had involved the entire left ear and a portion of the scalp. But after giving the full dose of the iron with the quinine four times, it was checked and faded away gradually. The parts were washed regularly after the second day from operation with the antiseptic solution of Gross, and dressed with carbolized cotton and vaseline.

Her principal diet was milk with an occasional eggnog and subsequently with beef extract.

By an accident my notes of the case from February 13th to March 10th were destroyed, and at the latter period, finding that the patient's upper teeth were much decayed, with their ragged

FIG. 2.

edges irritating the adjoining tissues, it was determined in consultation with Dr. Gaston to have them extracted. This was done under anesthesia by B. H. Catching, D. D. S., of this city, showing abscesses at the roots of two of them. March 13th, after a night of discomfort from the remaining teeth, I concluded to have them removed also; and after giving the A. C. E. mixture, Tom Crenshaw, D. D. S., extracted the nine other decayed teeth to her great relief. March 15th, the patient walked with some assistance nearly a quarter of a mile and had the accompanying photograph taken. March 16th, she walked without assistance to the Union depot, and boarding the train she departed for her home with the hope of complete recovery.

## Figures and Tables

**Fig. 1. f1:**
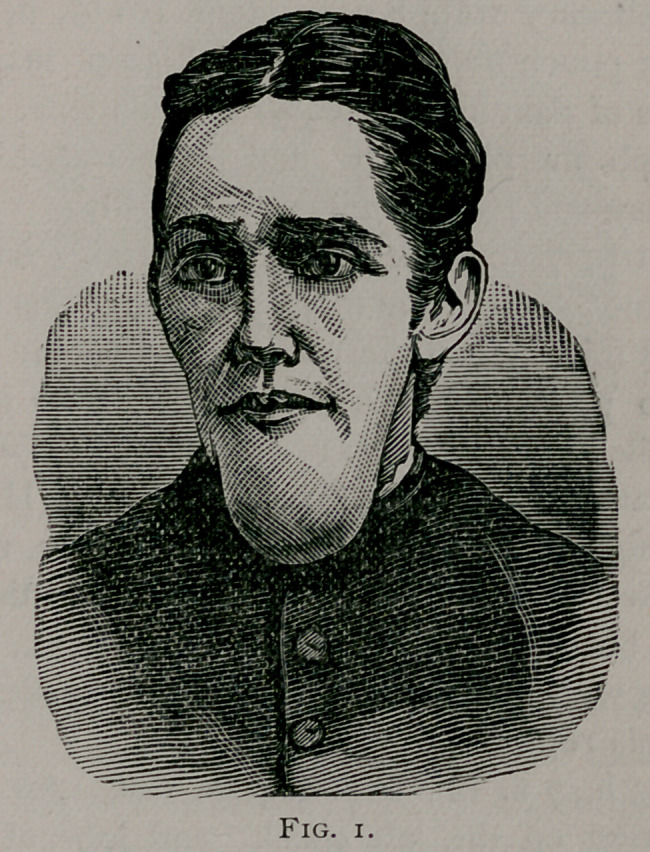


**Fig. 2. f2:**